# Why Do the Youths in Northeast India Use Tobacco?

**DOI:** 10.1155/2017/1391253

**Published:** 2017-05-31

**Authors:** Laishram Ladusingh, Preeti Dhillon, Pralip Kumar Narzary

**Affiliations:** ^1^Department of Mathematical Demography and Statistics, International Institute for Population Sciences, Mumbai, India; ^2^Department of Development Studies, International Institute for Population Sciences, Mumbai, India

## Abstract

This study is an assessment of the influence of parent's tobacco use on prospective tobacco use trajectories among young offspring. The study is based on unit level data from District Level Household and Facility Survey-4 (2012-2013) comprising 27,706 youths in 15–24 years' age group from northeastern states of India and used multilevel regression to identify the potential risk factors of tobacco consumption. The likelihood of using tobacco was found to be 3.4 and 1.14 times more, respectively, for the youths coresiding with mothers who use tobacco and fathers who use tobacco, in comparison to youths staying with parents not taking tobacco. The significant effect of peers on tobacco consumption among youths was also observed. School-going youths had significantly lower risk of tobacco use. The estimated likelihood of a young person from a household to use any tobacco, use smokeless tobacco, and smoke was found to be 28, 12, and 17 percent, respectively. There is an urgent need to extend National Tobacco Control Programme (NTCP) to the community level involving civil societies and young and adult generations for spreading awareness about the health hazards of tobacco use, providing support and facilitating quitting tobacco use.

## 1. Introduction

Most tobacco users started consumption of tobacco in their childhood and teens initially to show that they are mature and grown-up or accompany friends or experience the thrill of experimenting something forbidden or imitate their movie heroes and then they slowly get addicted to nicotine making it hard to quit afterwards despite repeated attempts. Nipping the problem in the bud, preventing youths from using tobacco products can effectively curtail tobacco use. Assessment of hurdles and searching for evidence-based policy inputs for amendment and strengthening of current policies to prevent tobacco use at young age are important from public health perspectives as smoking in young age is associated with incidence of asthma and chronic cough [1], cardiovascular disease, and cancer [2]. The Cigarettes and Other Tobacco Products Act (COTPA) of 2003 prohibits smoking in public places, prohibits sale of tobacco products to and by minors under 18 years, bans sale of tobacco products within 100 yards of all educational institutions, and made it mandatory to display pictorial fatal health warnings on tobacco products packages [3]. To strengthen implementation of COTPA, the Government of India enacted pilot-based National Tobacco Control Programme (NTCP) by setting up tobacco control cells at state levels for effective implementation and monitoring of antitobacco laws and initiatives [4]. The question is as follows: is COTPA effective in controlling tobacco use among teens and youths particularly in northeast India? The answer is a clear no, as evident from a study [5] that prevalence of smoking among students ranges from 34.5 percent in Mizoram to 19.7 percent in Assam and age at initiation of smoking is as low as 10 years, and prevalence of smoking among adults 15 years and older as in 2009-2010 was 35.4 percent and 10.7 percent in the aforesaid two states in the same order [6]. Based on a study of school-going children aged 11–19 years in Noida, Narain et al. (2013) [7] have found 4.1 percent of them to be currently using tobacco. Bagchi et al. (2014) [8] reported the high prevalence rate of 29.6 percent among adolescent students in Kolkata and age at initiation of smoking as 13.6 years. In the National Capital Territory (NCT), 5.4 percent of school children aged 13–18 years were found to be current tobacco users [9]. From an assessment of prevalence and correlates of tobacco use among 10–12-year-old students in Patna, Singh et al. (2005) [10] have found that 6 percent of the boys and 3.2 percent of girls were current tobacco users. Among school children in Jaipur, 2.1 percent of boys and 1.5 percent of the girls were reported as current tobacco users from a study of 10 randomly selected schools [11]. The aforesaid studies on tobacco use among school students have provided evidence that, despite provisions in COTPA and NTCP, tobacco use among children and adolescent is prevalent across India. This suggests the need for further investigation to find the most immediate factors outside the gambit of COTPA responsible for tobacco use among teens, adolescents, and youths. The paper aims to furnish more empirical evidence towards this objective and provide policy inputs which can complement the efforts of the government and civil societies to control the menace of tobacco use.

The specific objectives of this paper are to examine the trend in tobacco use including smoking and smokeless tobacco among youths in northeast (NE) India; explore household contextual factor influencing tobacco use among youths; find potential determinants of tobacco; and substantiate the findings in the sociocultural context of the region. The next section of the paper describes data and methods used for this study; it is followed by a section on results highlighting findings and its implications and the paper ends with a section on summary and discussion.

## 2. Materials and Methods

The present paper primarily uses data for northeast India from the District Level Household and Facility Survey fourth round conducted in 2012-2013 (DLHS-4, 2012-2013). DLHS-4 adopted a multistage stratified sampling design and covered seven states of northeast India (excluding Assam) [12]. Further, to examine the patterns of tobacco use, the study compiles the prevalence rates of tobacco use from Global Adults Tobacco Survey India (GATS India, 2009-2010) [6]. GATS India reported the prevalence of tobacco use among adults aged 15 years and above. For comparison purpose, we also estimate prevalence for the adults of same age group (15+) from DLHS-4. However, the remaining analysis is restricted to youth aged 15–24 using DLHS-4 data.

### 2.1. Variables Specification and Statistical Analysis

Among the possible determinants based on the literature review and the study objectives of tobacco use, we consider the age group, gender, place of residence, those currently attending school or not, wealth index of the household, and tobacco consumption status of mother and father. We create wealth index for northeastern states data using principal component analysis with 24 dummy variables on household's amenities and assets separately for rural and urban areas. Later it is categorized into low, medium and high-income group quartiles.

Based on the relationship with the head of the household and tobacco use status of individuals, we construct tobacco use among parents. A small sample of grandchildren of heads is considered as their children. Moreover, if parents of spouse and daughter/son in law did not reside in the same households, we take tobacco use status of their parents-in-law. We could not gather the information regarding parents of 1,297 (4%) youths who are other relatives or nonrelatives of the heads. Therefore, the final sample of the study is reduced to 27,706 youths who are in the age group of 15–24 years. We only consider sample for the usual residents in the households. Finally, mothers and fathers' use of any tobacco, use of smokeless tobacco, and smoking behaviour is linked with the children data. These parental level variables are in binary form: 1. if they are currently using tobacco; 0, if they are not using or they died or they are not usual residents. If a person consumes (1) pan with tobacco or (2) Gutka/pan masala with tobacco or (3) tobacco only, a person is considered as a user of tobacco, whereas usual smoker (at least once every day) or occasional smoker is considered as a current smoker. Hence, a person who currently either chews tobacco (any one of the above-mentioned items) or smokes is considered as using “any tobacco.”

The paper applies bivariate and multivariate analysis including two-level random intercept logistic regression models to identify the potential risk factors of tobacco use among the youths in northeast India. Whole analysis has been carried out using STATA (version-13, StataCorp LLC). For multilevel regression, we adopt MCMC method of estimation.

## 3. Results

Levels and trends of any tobacco use among adults above 15 years for the states of northeast (NE) India are shown in Table 1. Out of seven surveyed states in 2012-2013, in the four states of Meghalaya, Manipur, Tripura, and Mizoram, more than 50 percent of the adults reported using any tobacco. The levels of any tobacco use in these states have remained persistently high at above 50 percent during 2009-2010 to 2012-2013. Meghalaya and Manipur show the increase in tobacco use among women. On the other hand, Sikkim, Arunachal Pradesh, and Nagaland exhibit some decline in tobacco use owing to declining tobacco use among women.

Table 2 presents the statewide prevalence of any tobacco use, smoking, and smokeless tobacco among the youths aged 15–24 years in northeast India by sex. In the states of Mizoram, Meghalaya, Nagaland, and Manipur, more than one-third of youths are found to be using some or the other forms of tobacco. The largest proportions of youths using any tobacco are found in Meghalaya (54% among boys and 30% among girls) and Mizoram (53% among boys and 37% among girls). Considerable sex differential in smoking among youths is found in most states in northeast India. Smoking among male youth ranges between 14% in Tripura and 47% in Mizoram and among female youth between 1% in Tripura and Sikkim to 8% in Mizoram. The level of smokeless tobacco use is much higher than that of the level of smoking in most states of northeast India. Mizoram, Meghalaya, and Tripura are the northeastern states where more girls consumed smokeless tobacco than boys. Overall, the higher levels of tobacco consumption in northeast states show that the young population of northeast states is at higher risk of tobacco-associated diseases.

### 3.1. Factors Affecting Tobacco Use among Youths

**Figure 1:**
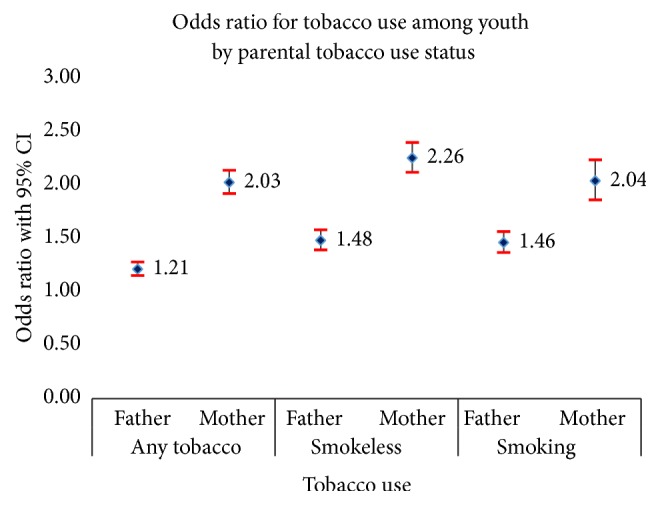
Odds ratios and 95 percent confidence interval of youths' tobacco use by parental tobacco use.

Odds ratios from logistic regression with 95% confidence interval (CI) are shown in Figure 1, to explore household context of grooming tobacco use among youths. It is noted that odds ratio for any tobacco use is 1.21 (95% CI: 1.15, 1.28) for youth whose father used tobacco and 2.03 (95% CI: 1.92, 2.14) for those whose mother consumes tobacco. Moreover, youths whose father and mother are using smokeless tobacco are 1.48 (95% CI: 1.39, 1.58) and 2.26 (95% CI: 2.12, 2.40) times, respectively, more likely to use smokeless tobacco compared to whose father and mother do not consume. Similarly, youths whose father and mother smoke are 1.46 (95% CI: 1.37, 1.57) and 2.04 (95% CI: 1.86, 2.24) times more likely to smoke compared to whose father and mother do not smoke.

Results of multilevel logistic regressions depicting the effects of parental tobacco use behaviour and household level context on tobacco use among youths are shown in Table 3. From the null model that is without any covariate, the odds of using any tobacco among youth in a household is 0.32, and the corresponding probability is 0.24. Similarly, odds of using smokeless tobacco and smoking in a household are 0.13 and 0.15, respectively, and corresponding probabilities are 0.12 and 0.13. Nearly 40% of the variation in tobacco use among the young population is explained by the variation in tobacco use between households. Further, this intraclass correlation coefficient (ICC) is higher for any tobacco use (50%) than smoking (20%).

After controlling for socioeconomic factors and parental use of tobacco, the model estimates 0.38 odds ratio for using any tobacco in a household. The probability of using tobacco in a household is 28% for any tobacco use, 12% for smokeless tobacco, and 17% for smoking. After controlling other factors, nearly half of the variation in tobacco use among the young population is explained by the variation between households only. This suggests the significant clustering of tobacco use within households. In other words, young population is influenced by the tobacco use behaviour of their peers residing in the households. Further, this coefficient is higher for both smokeless tobacco use (54%) and smoking (39%).

Youths aged 20–24 years are 2.8 times more likely to use any tobacco and 2.3 and 2.6 times more likely to use smokeless tobacco and smoke in comparison to 15–19-year-old persons. Female youths significantly have a lower risk of using any tobacco compared to male counterparts. However, this gender difference is not huge in terms of smokeless tobacco consumption. Youths who have been attending school have significantly lower risk of tobacco use as their odds of tobacco use is 0.22 (*p* < 0.001) for any tobacco, 0.30 (*p* < 0.001) smokeless tobacco, and 0.28 (*p* < 0.001) for smoking. Place of residence in rural or urban does not make any significant difference in risk of tobacco use. Youths from wealthy households as measured by wealth quintile have a lower risk of tobacco use as compared to their peers in lower wealth quintile.

One of the interesting outcomes of the paper is that there is a contextual effect of the parental use of tobacco on their children tobacco use behaviour. The findings suggest that the youths who live with their mothers who have been using any tobacco are at 3.4 times more risk of using tobacco, similarly, those who live with mothers using smokeless tobacco have 1.59 times more chance to use smokeless tobacco, and those who live with the smoking mother are 2.52 times more likely to smoke. On the other hand, youths who live with their fathers who consume any tobacco are 1.14 times more likely to use any tobacco, and those who live with fathers who have been smoking have 1.54 times more risk to smoke than whose fathers have not been smoking.

## 4. Summary and Discussion

DLHS is conducted with the aim of providing district level estimates for various indicators with a large sample size. It allows robust statistical analysis to investigate phenomena covered under the survey. As such, it is quite appropriate to use DLHS data to assess the tobacco use behaviour in the form of smoking and smokeless tobacco and its dual use that has important health policy implications. Present study focused on youths of northeast India, because the prevalence of tobacco use is much higher than the national average in all the states of the region. As per the NFHS 2005-06 [13] and GATS 2009-10 [6] report, the percentage of tobacco use in all the eight northeastern states is much higher than the national average for both male and female. Further, tobacco use (of any kind) in northeast India is consistently high in DLHS-4. These strongly affirm that tobacco use is socially acceptable and integral part of the culture of the region. The trend in tobacco use also suggests that COPTA is not effective in the region and alarm bell is ringing to save the soul of the youths. Use of tobacco among youths has serious health and social implications. They are going to use it for a longer period until they reach the feeble stage, or if there is some health catastrophe because old habit dies hard. The youths have to bear the brunt of passive tobacco use for a longer duration. Further, while they reach adulthood and become responsible citizens, they may not reprimand youngsters for using tobacco or in the worst case they may even encourage them to use tobacco. Among the youths (15–24 years), smokers are more than the users of smokeless tobacco in Sikkim, Arunachal Pradesh, and Mizoram. Further, a distinct gender differential has emerged in the pattern of the use of tobacco. Male youth smoker is more than smokeless tobacco users in Sikkim, Arunachal Pradesh, Meghalaya, and Mizoram. But among females, the user of smokeless tobacco is much higher than smokers in all the states.

In the states where adult tobacco use is high, the percentage of tobacco use among youth is also high. It suggests that use of tobacco among adults has a profound effect on the youth tobacco use behaviour. Further, the effect of parents' tobacco use on youth is crystal clear. Other studies [5, 8] also found a positive relationship between parental use of tobacco on children's behaviour. This linkage is because the young children and youth tend to imitate or follow their elders by observing them. The present study indicates that the behaviour of the mother seems to matter much more than that of father's in terms of youth's behaviour. We postulate that this might be because children are usually more attached to their mothers and, as mothers spend more time at home, the children observe her behaviour more closely. On the other hand, as fathers spend most of the day away from home, the impact of his behaviour on children appears to be weaker and sometimes might even go unnoticed. Further, parents' use of tobacco may give easy access to youth to such substances

COPTA prevents the sale of any tobacco product within 200 meters of educational institution and sale to minors. However, implementation of COPTA alone may not be able to reduce the use of tobacco among the youths, unless the behaviour of the youth is monitored at the household level. Some of the ways to reduce tobacco use among the youths are to regulate the production of tobacco and finished tobacco products and their supply. Another way is to raise the price of tobacco products multiple times so that youths find it difficult to afford. As tobacco use is culturally accepted behaviour in the region, especially among the adults, the policy needs to address it from this perspective. Another study [14] also suggests the need to take tobacco control programme beyond the regulation of banning sales of tobacco products within 200 meters of academic institution and banning of sales of tobacco to minors. The public programme alone is not sufficient to ward off tobacco use and community's involvement is crucial for making any programme effective.

## Figures and Tables

**Table 1:** Levels and trends of any tobacco use among adults above 15 years in northeast India.

States in NE India	GATS India (2009-2010)			DLHS-4 (2012-2013)		
	Total	M	F	Total	M	F
Sikkim	41.6	48.7	33.2	27.8	41.0	16.2
Arunachal Pradesh	47.7	64.0	31.7	35.5	55.1	17.6
Nagaland	56.8	69.2	43.0	41.2	59.1	23.2
Meghalaya	55.2	73.2	36.7	51.9	66.0	42.8
Manipur	54.1	66.6	41.2	53.8	67.8	41.8
Tripura	55.9	63.4	48.1	56.1	63.0	49.5
Mizoram	67.2	72.5	61.6	65.4	74.0	57.4
Assam	39.3	52.6	25.3	NA	NA	NA

*Note*. GATS: Global Tobacco Adults Survey; DLHS: District Level Household and Facility Survey.

**Table 2:** Mode of tobacco use by youths (15–24 years) in northeastern states of India, 2012-2013.

	Any tobacco use			Smoking			Smokeless tobacco use		
	Total	Males	Females	Total	Males	Females	Total	Males	Females
Sikkim	12.6	24.6	3.5	9.1	19.6	1.2	6.2	11.3	2.3
Arunachal Pradesh	19.7	33.6	8.2	14.2	26.8	3.9	11.5	18.4	5.8
Nagaland	33.6	46.2	23.3	11.0	22.2	1.9	30.9	40.7	23.0
Meghalaya	39.6	53.9	29.9	17.9	39.8	3.1	27.4	25.3	28.8
Manipur	33.5	42.8	26.3	13.6	24.0	5.6	28.7	35.0	23.8
Tripura	29.6	31.6	28.0	7.0	13.6	1.3	26.1	24.7	27.3
Mizoram	44.3	52.9	36.6	26.7	47.4	8.1	24.4	16.1	31.8

*Note*. The figures are in percent.

**Table 3:** Risk factors of tobacco use among youths in northeast India, 2012-2013.

	Any tobacco			Smokeless tobacco			Smoking tobacco		
	Odds ratio	[95% CI]		Odds ratio	[95% CI]		Odds ratio	[95% CI]	
*Null model*									

Constant	0.32^*∗∗∗*^	0.30	0.33	0.13^*∗∗∗*^	0.12	0.14	0.15^*∗∗∗*^	0.14	0.16
Household level									
Var(cons)	2.23	1.97	2.51	3.33	2.93	3.80	0.84	0.63	1.08
Probability	0.24			0.12			0.13		
ICC	0.40			0.50			0.20		

*Models with explanatory variables*									

Constant	0.38^*∗∗∗*^	0.33	0.43	0.14^*∗∗∗*^	0.12	0.17	0.21^*∗∗∗*^	0.18	0.23
Age									
15–19									
20–24	2.84^*∗∗∗*^	2.57	3.13	2.30^*∗∗∗*^	2.05	2.55	2.59^*∗∗∗*^	2.31	2.92
Gender									
Male									
Female	0.19^*∗∗∗*^	0.17	0.21	0.62^*∗∗∗*^	0.56	0.67	0.05^*∗∗∗*^	0.04	0.06
Attending school									
No									
Yes	0.22^*∗∗∗*^	0.20	0.25	0.30^*∗∗∗*^	0.26	0.34	0.28^*∗∗∗*^	0.24	0.32
Place of residence									
Urban									
Rural	1.01	0.92	1.11	0.99	0.89	1.11	1.15^*∗∗*^	1.03	1.28
Wealth index									
Low									
Medium	0.89^*∗*^	0.79	0.99	0.91^*∗*^	0.81	1.01	0.87^*∗∗*^	0.77	0.98
High	0.78^*∗∗∗*^	0.70	0.87	0.71^*∗∗∗*^	0.62	0.79	0.86^*∗∗*^	0.76	0.96
Mother use of tobacco	3.44^*∗∗∗*^	3.10	3.84	1.59^*∗∗∗*^	1.35	1.86	2.52^*∗∗∗*^	2.17	2.93
Father use of tobacco	1.14^*∗∗*^	1.04	1.25	1.08	0.95	1.20	1.54^*∗∗∗*^	1.38	1.71

Level of household									
Var(cons)	3.26	2.94	3.66	3.88	3.38	4.40	2.11	1.78	2.43

Probability	0.28			0.12			0.17		
ICC	0.50			0.54			0.39		

*Note*. The probability of using tobacco in the average household. ICC: intraclass correlation coefficient, ^*∗∗∗*^*p* < 0.001, ^*∗∗*^*p* < 0.01, ^*∗*^*p* < 0.05.
